# Genome-wide association study of drought tolerance in wheat (*Triticum aestivum* L.) identifies SNP markers and candidate genes

**DOI:** 10.1007/s00438-024-02104-x

**Published:** 2024-03-02

**Authors:** Sina Nouraei, Md Sultan Mia, Hui Liu, Neil C. Turner, Guijun Yan

**Affiliations:** 1 UWA School of Agriculture and Environment, The University of Western Australia, Perth, WA 6009 Australia; 2 The UWA Institute of Agriculture, The University of Western Australia, Perth, WA 6009 Australia; 3 Department of Primary Industries and Regional Development, 3 Baron-Hay Court, South Perth, WA 6151 Australia

**Keywords:** Association mapping, Genetic structure analysis, Linkage disequilibrium, Stress susceptibility index (SSI), Stress tolerance index (STI)

## Abstract

Drought stress poses a severe threat to global wheat production, necessitating an in-depth exploration of the genetic basis for drought tolerance associated traits. This study employed a 90 K SNP array to conduct a genome-wide association analysis, unravelling genetic determinants of key traits related to drought tolerance in wheat, namely plant height, root length, and root and shoot dry weight. Using the mixed linear model (MLM) method on 125 wheat accessions subjected to both well-watered and drought stress treatments, we identified 53 SNPs significantly associated with stress susceptibility (SSI) and tolerance indices (STI) for the targeted traits. Notably, chromosomes 2A and 3B stood out with ten and nine associated markers, respectively. Across 17 chromosomes, 44 unique candidate genes were pinpointed, predominantly located on the distal ends of 1A, 1B, 1D, 2A, 3A, 3B, 4A, 6A, 6B, 7A, 7B, and 7D chromosomes. These genes, implicated in diverse functions related to plant growth, development, and stress responses, offer a rich resource for future investigation. A clustering pattern emerged, notably with seven genes associated with SSI for plant height and four genes linked to both STI of plant height and shoot dry weight, converging on specific regions of chromosome arms of 2AS and 3BL. Additionally, shared genes encoding polygalacturonase, auxilin-related protein 1, peptide deformylase, and receptor-like kinase underscored the interconnectedness between plant height and shoot dry weight. In conclusion, our findings provide insights into the molecular mechanisms governing wheat drought tolerance, identifying promising genomic loci for further exploration and crop improvement strategies.

**Graphical Abstract:**

(1) A diverse panel of wheat genotypes was cultivated under both well-watered and drought stress conditions; (2) Phenotyping involved washing, scanning, drying and weighing plants to evaluate the stress susceptibility (SSI) and stress tolerance (STI) indices for four drought tolerance-related traits; (3) Genotyping was performed by extracting DNA and using the wheat 90 K Illumina iSelect array; (4) Phenotypic and genotypic data were utilized in a genome-wide association analysis (GWAS) using a mixed linear model (MLM); (5) Population structure assessment, principal component analysis (PCA), and kinship analysis were conducted; (6) Candidate genes were identified, and (7) their molecular functions were analysed and discussed.

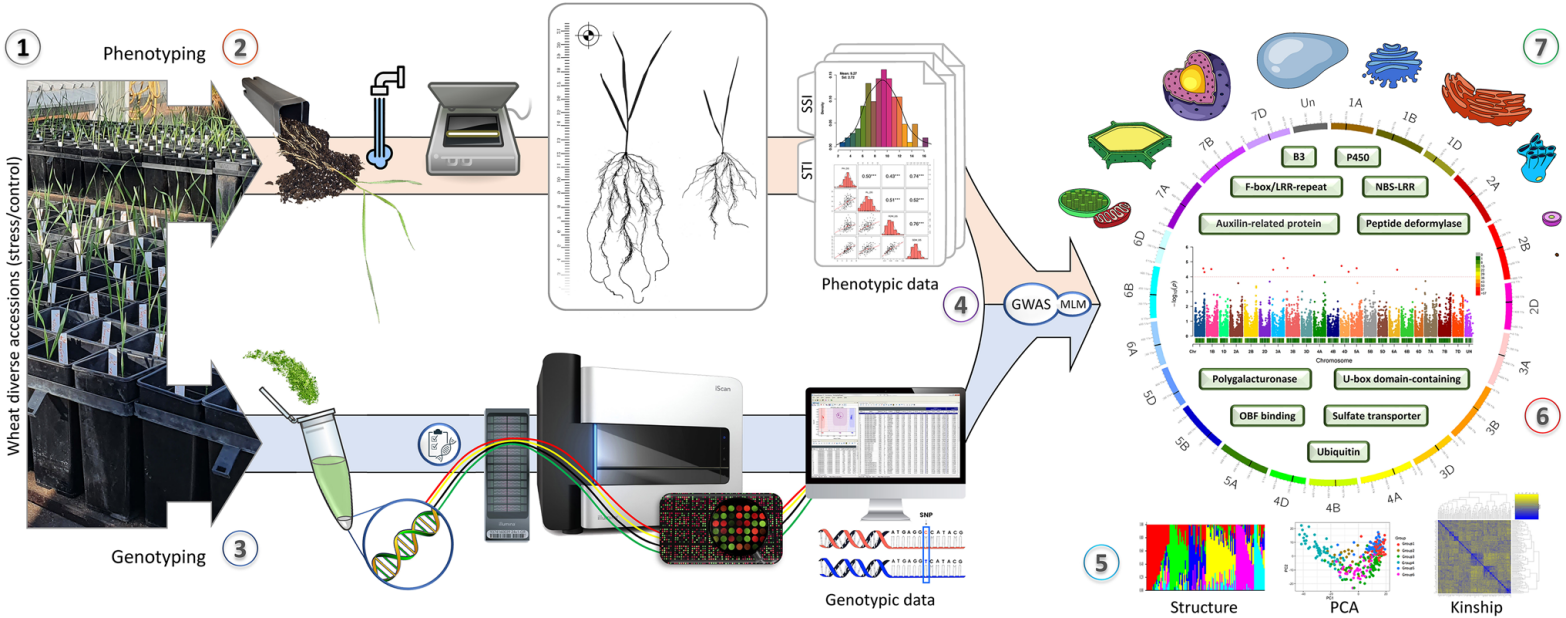

**Supplementary Information:**

The online version contains supplementary material available at 10.1007/s00438-024-02104-x.

## Introduction

Wheat (*Triticum aestivum* L.), in the family Poaceae, originated in West Asia and is cultivated throughout the world (Ahmed et al. 2022). It is an important source of carbohydrates, proteins, and fiber for approximately one-third of the global population (Grote et al. 2021). Global food demand is growing rapidly and is expected to double by 2050 (Tilman et al. 2011). Meanwhile, climate change-induced drought and heat stresses are endangering global food productivity and food security (Hatfield and Dold 2018). The projections show that feeding a world population of 9.1 billion people will require raising overall wheat production by 60% by 2050 (Jaggard et al. 2010). To keep up with this demand, wheat cultivars with enhanced yield potential and improved tolerance to biotic and abiotic stresses need to be developed.

Drought stress is one of the major constraints of wheat production which can occur at any growth stage, depending on the geographical location (Sallam et al. 2019). The impact of drought on wheat can vary from 10 to 90% of its potential yield, depending on drought intensity, duration of drought, and growth stage of the plant (Farooq et al. 2014). The three stages of seedling establishment, booting and grain filling are the most sensitive growth phases for drought stress (Fischer and Turner 1978). Drought at the vegetative stage reduces plant height, leaf surface area, and root and shoot biomass production while causing pollen sterility and reducing grain number and weight at the reproductive stage (Hussain et al. 2008; Shokat et al. 2020). The study of terminal drought tends to receive most attention in wheat, due to the high sensitivity of this stage. However, drought stress at the vegetative stage can also significantly affect the overall yield potential of plants (Sallam et al. 2018).

Some studies have investigated the impact of drought at an early growth stage on final plant performance. Drought at the vegetative stage can have a significant impact on the yield of wheat by reducing shoot biomass and the number of fertile spikes produced at the establishment and tillering stages (Ahmad et al. 2022). A close correlation between drought tolerance for seedling dry weight and grain yield has been reported in wheat, triticale, and maize (Grzesiak et al. 2012; Dodig et al. 2015). The root is also one of the components of wheat affected by drought stress along with soil conditions. Root biomass and length at the seedling stage are vital genetic traits for final plant performance under water-deficit condition. A high root length can improve the adaptation of genotypes to water stress by providing better access to moisture deep in the soil and result in more vigorous seedling establishment (Ahmed et al. 2019).

Characterization of plant morphological and physiological traits associated with drought tolerance is essential for evaluation and selection of desirable drought tolerant genotypes (Khan et al. 2016). However, the difficulty of direct selection for drought tolerance necessitates adoption of indirect selection criteria to dissect the complex set of interrelated traits which make up drought tolerance. Stress tolerance index (STI) and stress susceptibility index (SSI) are two reliable indices that have been widely used for assessing plant performance under drought conditions (Khanzada et al. 2020). Each of these measures has a distinct perspective on drought tolerance and thus employing both indices should deliver an accurate evaluation of drought tolerance (Wu et al. 2022).

For breeding drought-tolerant genotypes, it is important to understand the mechanisms and responses of plant to water shortage. At a physiological level, wheat reduces water losses by stomatal closure which simultaneously reduces the rate of photosynthesis and leads to a decrease in CO_2_ fixation and nicotinamide adenine dinucleotide phosphate (NADP +) as the final acceptor of the electron in electron transport chain (Camaille et al. 2021). The leaked electrons to O_2_ result in overproduction of reactive oxygen species (ROS) that induce oxidative damage in plant tissues (Cruz de Carvalho 2008). The molecular response of wheat to water shortage involves a series of pathways for signal reception, transduction, gene expression and production of stress metabolites (Wu et al. 2022). The genes that are induced by drought stress can be classified into two main groups. The first group includes genes whose products directly play role in stress tolerance, such as genes encoding late embryogenesis abundant proteins (LEA) and chaperones, osmolytes such as proline and glycine betaine, and detoxification enzymes like catalases, proteases, and peroxidases (Dash et al. 2014). The second group comprises genes that modulate the expression of stress responsive genes as well as playing a role in signal transduction, such as different transcription factors (TFs), transcriptional regulators (TRs) and protein kinases (PKs) (Lata et al. 2015).

Molecular breeding for plant drought tolerance has become a hot research area in recent years. It is an effective and economic approach for coping with drought stress since the genes introduced into the breeding lines are heritable (Rauf 2008). Drought tolerance is a complex quantitative trait controlled by many micro-effective genes and highly influenced by genotype by environment interactions (Khan et al. 2019). Many studies have been conducted to date for deciphering the molecular basis of drought tolerance in wheat at different growing stages (Kirigwi et al. 2007; Alexander et al. 2012; Tahmasebi et al. 2017; Zandipour et al. 2020). However, previous studies were mostly based on linkage analysis of recombinant inbred line populations (RILs) derived from crosses between two parents with significantly different phenotypes. Bi-parental QTL mapping suffers from some limitations. First, a RIL population has few recombination events and can only be used to detect genes that show a significant difference between parental lines (Korte and Farlow 2013). Second, a large proportion of the studies have been based on low-resolution molecular maps consisting of only 100–1000 SSRs, EST-STS, and DArT markers, insufficient to saturate the large 17 giga-base-pair wheat genome (Rabbi et al. 2021).

Genome wide association study (GWAS) is a powerful method designed to identify genotype–phenotype associations by evaluation of the genetic variants across the genomes of many individuals (Pu et al. 2022). Compared to linkage analysis, GWAS uses a natural population, thereby saving a lot of time by eliminating the needs for population construction (Alqudah et al. 2020). Additionally, by employing high resolution markers and natural populations with high diversity, GWAS can identify more loci responsible for the traits compared to bi-parental QTL mapping (Liu et al. 2022). The diverse and unstructured natural populations used in GWAS have allowed the accumulation of a large amount of information on historical recombinations and thus deliver a relatively high mapping resolution (Alqudah et al. 2020). In recent years, association studies have been extensively used for the genetic dissection of drought tolerance in several crops, such as wheat (Qaseem et al. 2019), *Arabidopsis* (Bac-Molenaar et al. 2016), barley (Wehner et al. 2015), and rice (Ma et al. 2016). However, only a few genome-wide association studies have been conducted in wheat to genetically dissect mechanisms of drought tolerance at the seedling stage.

In this study, we evaluated 125 wheat accessions at the seedling stage under well-watered and drought-stress conditions, employing a 90 K SNP array for GWAS on four drought tolerance-related traits, along with two stress indices (SSI and STI). Our objectives were to (1) investigate phenotypic variations among accessions under different water availabilities, (2) identify genomic regions associated with drought tolerance at the seedling stage based on important phenotypical traits, and (3) unveil key biological processes and pathways of genes associated with crucial drought-tolerance traits. This knowledge deepens our understanding of underlying molecular mechanisms and provides a roadmap for developing precise crop improvement strategies. The identified genes can offer targets for molecular breeding and biotechnological interventions aimed at developing drought-tolerant wheat varieties.

## Materials and methods

### Plant materials

A panel of 125 accessions of wheat from 15 countries around the world was used for genome-wide association analysis (Supplementary Table S1). Seeds were sourced from the Australian Winter Cereals Collection and wheat breeding companies/institutions including InterGrain Pty Ltd., Australian Grain Technologies, LongReach Plant Breeders, Edstar Genetics Pty Ltd., Chinese Academy of Agriculture Sciences, Inner Mongolia Academy of Agriculture and Animal Husbandry Science, and Gansu Academy of Agricultural Sciences. Some of the accessions in this panel were shown by Ayalew et al. (2015) to have a wide range of variability for early-stage water stress tolerance.

### Experimental design and treatments

A randomized complete block design (RCBD) experiment with three biological replications was conducted in June 2022 in a glasshouse facility at The University of Western Australia in Perth, Western Australia (31°59′S, 115°49′E). Square plastic pots 8 cm × 8 cm × 18 cm were filled with 800 g air-dried potting mix containing brown river sand (60%) and fine cocopeat (40%). The pots were watered to 100% pot capacity (PC) by watering until free draining and then allowing the pots to drain for 48 h before weighing the pots and sowing the seeds at 2 cm depth (Turner 2019). After emergence, two water treatments were imposed, (i) well-watered (WW) and (ii) drought stress (DS). In WW, the pots were kept between 80 and 100% PC by regular weighing and watering. In DS, pots were irrigated once after germination and then allowed to dry without any further watering until the end of the experiment. Plants were harvested 15 days after germination when the soil water content was on average 93% PC in WW and 26% PC in DS. The plants were gently removed from the pots before gently washing the roots with tap water to completely remove the remaining soil medium.

### Phenotypic evaluation and statistical analysis

Plant height (PH), root length (RL), root dry weight (RDW), and shoot dry weight (SDW) were measured for each seedling. PH was measured by ruler from the base of the stem to the tip of the longest leaf. For RL, the root sample was placed in a glass rectangular tray (20 cm × 15 cm) with a 4–5 mm layer of water to untangle the roots and minimize root overlap, as previously described by Peng et al. (2010), and scanned by Epson V850 Pro Scanner (Epson, Tokyo, Japan). Epson scan software v3.9.3 (Epson, Tokyo, Japan) was used with a scanning setting of 8-bit grayscale and 400 dots per inch (dpi) resolution. The root scan images were analysed in RhizoVision Explorer v2.0.3 (Seethepalli and York 2020) using algorithms described by Seethepalli et al. (2021) to obtain the total root length per seedling. For RDW and SDW, leaf and root samples were dried in a 60 ℃ oven for 72 h before being weighed with an analytical balance accurate to 0.1 mg. To evaluate the stability of the phenotypic traits in response to drought stress compared to the WW treatment, the SSI and STI were calculated for each trait as follows:

$${\text{SSI}}= \frac{1-\frac{{Y}_{si}}{{Y}_{pi}}}{1-\frac{{\overline{Y}}_{si}}{{\overline{Y}}_{pi}}}$$

$${\text{STI}}= \frac{{Y}_{si}\times {Y}_{pi}}{{{\overline{Y}}_{pi}}^{2}}$$

where Ysi = performance of a genotype in the DS treatment; Ypi = performance of the same genotype in the WW treatment; Y̅si = mean Ysi of all genotypes, Y̅pi = mean Ypi of all genotypes (Fischer and Maurer 1978). The genotypes with lower SSI and higher STI values for a trait, showed less reduction in that trait as a result of drought stress comparing to the WW treatment (i.e., higher stability for that trait) (Wehner et al. 2015).

The analysis of variance (ANOVA) was carried out for the phenotypic data to determine statistical differences among accessions in response to drought stress by the statistical model${:y}_{ij} = \mu + {\tau }_{i} + {\beta }_{j} + {\upvarepsilon }_{ij}$, where yij is the observed value, μ is the overall mean, τi the effect of the ith genotype, βj the effect of the jth block, and εij is random error. Descriptive statistics of traits were calculated with SPSS v29 (SPSS, Chicago, IL, USA). The Kolmogorov–Smirnov and Shapiro–Wilk tests were performed to validate the normality of the phenotypic data using SPSS software v29 (SPSS, Chicago, IL, USA). The frequency distributions and Pearson’s correlation coefficients of the traits in individual environments were obtained using R package ggplot2 v3.4.1 (Wickham 2016) and psych v2.2.9 (Revelle 2023) in R v4.2.2 software (R Core Team 2020).

### Wheat 90 K SNP Illumina iSelect genotyping

Leaves of seedlings were harvested at 3-leaf stage. DNA of each accession was extracted from the leaves using the cetyl trimethyl ammonium bromide (CTAB) method and stored in TE buffer (Wang et al. 2019b). The quantity, purity and integrity of the extracted DNA was checked by NanoDrop 2000 (Thermo Fisher Scientific Inc., CA, USA) and agarose gel electrophoresis. The accessions were genotyped using the wheat 90 K Illumina iSelect array and analysed by genome studio software v2.0 (Illumina Inc., CA, USA), following the protocol described by Wang et al. (2014), which generated 51,426 SNP markers. After filtering and excluding SNPs with > 0.25 heterozygous calls and minor allele frequency (MAF) ≤ 5%, and genotypes with missing data > 20%, a total of 36,586 SNPs were retained and used for the GWAS analysis.

### Population structure and linkage disequilibrium analysis

The population structure was analysed in STRUCTURE v2.3.4 based on the Bayesian clustering model (Falush et al. 2003). The number of subpopulation groups (K) was predefined from two to nine with five times iteration for each K value, which was run with 10,000 MCMC (Markov-Chain Monte Carlo) replicates and 10,000 burn-in periods. The STRUCTURE output was visualized in STRUCTURE HARVESTER web-based program (http://taylor0.biology.ucla.edu/structureHarvester/) and the number of K groups that best fit the dataset was determined according to the ΔK calculated by the Evanno method (Evanno et al. 2005; Earl and vonHoldt 2012).

Linkage disequilibrium (LD) of individual chromosomes, three sub-genomes, and the whole genome was calculated by measuring the squared allele frequency correlations (*r*^2^) (VanLiere and Rosenberg 2008) between pairs of SNPs in TASSEL 5.2.86 software (Bradbury et al. 2007), with a sliding window of 50 markers. Using TASSEL output, the *r*^2^ values were plotted against the genetic distance and the locally-weighted polynomial regression (LOESS) curve was drawn to determine the LD decay by a custom R script in R v4.2.2 software (R Core Team 2020). LD decay was identified as the physical genomic distance at which the *r*^2^ decreased to half of its maximum value, where *r*^2^ = 1 indicating complete LD, and *r*^2^ = 0, indicating absence of LD.

### GWAS analysis (association mapping)

A total of 125 genotypes and 36,586 SNPs were used for GWAS analysis in TASSEL 5.2.86 software (Bradbury et al. 2007) to map associations between SSI and STI indices of phenotypic traits and SNP markers. Initially, the LD kNNi algorithm was implemented for imputation of the missing data in the genotypic file with default setting. Principle components analysis (PCA) was carried out and a kinship matrix was created from the genotypic data in TASSEL. Phylogenetic trees were constructed using the neighbour-joining method in the iTOL server (https://itol.embl.de/) (Letunic and Bork 2021). GWAS analysis was carried out using a mixed linear model (MLM) in TASSEL, and population structure and kinship coefficients were taken into account to avoid false associations. The Manhattan plot was used to demonstrate the correlation between SNP and phenotypic traits. The quantile–quantile (Q-Q) plot was used to show the level of difference between observed and predicted values. The Manhattan plots and Q-Q plots were constructed from TASSEL output using R package rMVP (Yin et al. 2021) in R v4.2.2 software (R Core Team 2020). The Bonferroni correction threshold − log10 (*p*) > 5.86 (*p* = 0.05/N; N = total markers used) was too stringent in this study. Therefore, less strict *p*-value thresholds of − log10 (*p*) > 4 and − log10 (*p*) > 5 were set for the identification of true marker-trait association in the Manhattan plots (Chen et al. 2021c). The identified markers were mapped and visualised on an idiogram using R package RIdeogram (Hao et al. 2020).

### Identification of candidate genes

The SNP markers significantly associated with the traits were searched in JBrowser 1.16.3 (https://urgi.versailles.inra.fr) against the wheat reference genome IWGSC RefSeq v1.0 (The International Wheat Genome Sequencing et al. 2018) to find the physical position of the identified markers and flanking genes. A gene with a marker located within it or the closest high-confidence gene within 2 Mbp flanking of the SNP’s physical position was considered as the associated gene to that marker. Next, the gene ID was searched in Ensembl Plants database (https://plants.ensembl.org/Triticum_aestivum/Info/Index) to find the annotation of the genes. The complementary information on molecular function of the identified genes was extracted from UniProt (https://www.uniprot.org), InterPro (https://www.ebi.ac.uk/interpro), and PANTHER databases (http://www.pantherdb.org).

### In silico expression analysis of the identified genes

The wheat multi-omics database (WheatOmics, http://202.194.139.32/expression/wheat.html) was used to investigate the expression of the identified genes in previous studies (Ma et al. 2021b). The relative difference between gene expression in tolerant and susceptible genotypes was determined using the formula:

$$\mathrm{Relative \,\, Difference }(\%)=\left|\frac{A-B}{\frac{A+B}{2}}\right|\times 100$$

Here, A and B represent the two values being compared. The numerator of the formula represents the absolute difference between A and B, while the denominator takes into account their average. The resulting percentage provides a measure of how closely the two values are related. The average of the relative expression differences across all genes was calculated to illustrate the overall expression status of genes in tolerant and susceptible genotypes in the WW and DS treatments.

## Results

### Phenotypic response to drought stress

**Fig. 1:**
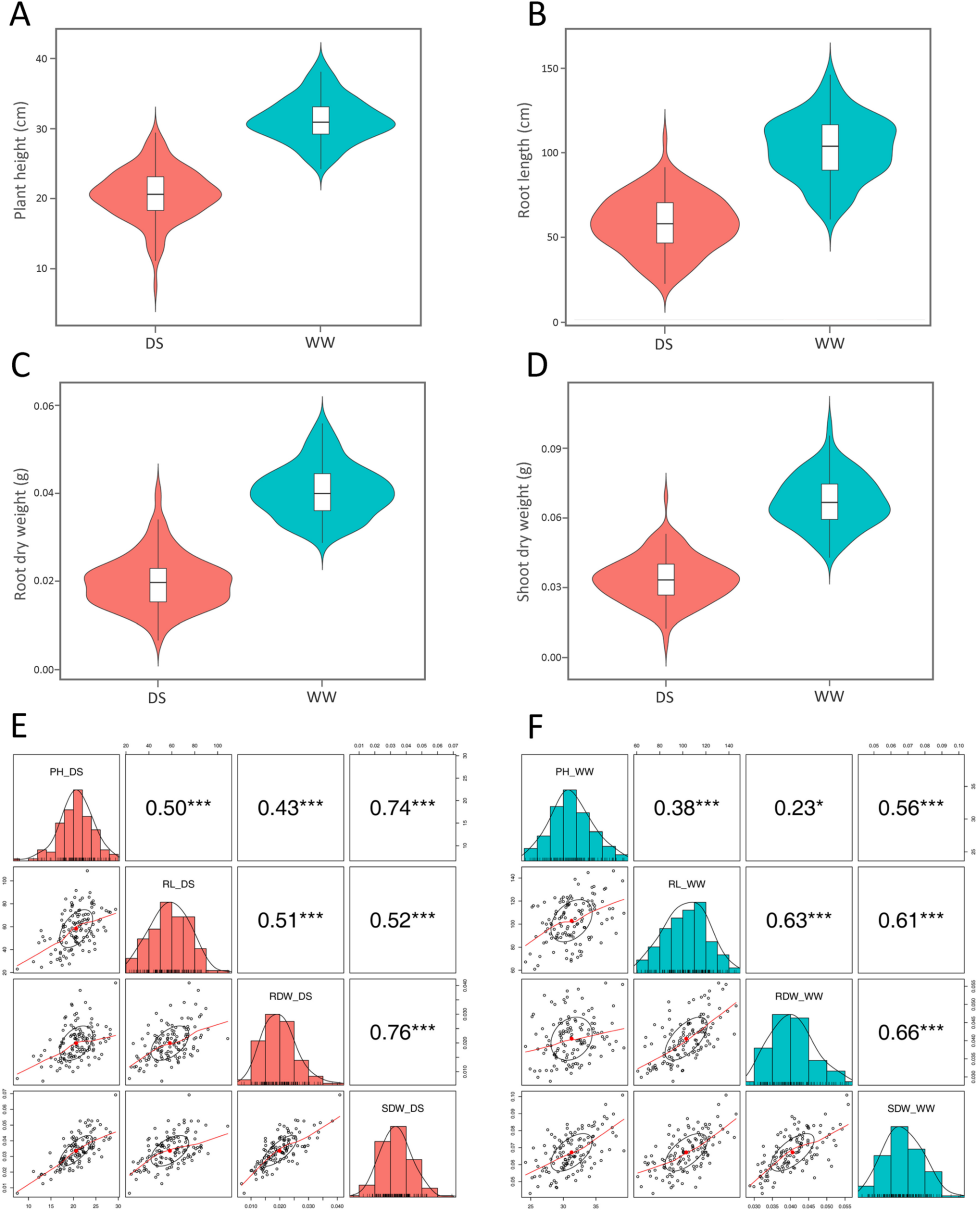
Violin plots showing frequency distribution of (**A**) plant height, (**B**) root length, (**C**) root dry weight, and (**D**) shoot dry weight of 125 wheat genotypes given two water treatments: drought stress (DS, red) and well-watered (WW, green). Pearson’s correlation analysis visualising correlations between the seedling traits in the (**E**) drought-stressed and (**F**) well-watered treatments; PH, plant height; RL, root length; RDW, root dry weight; SDW, shoot dry weight. Significant levels are * *p* < 0.05, ** *p* < 0.01, and *** *p* < 0.001

ANOVA analysis showed significant differences (*p* < 0.01) among genotypes for the phenotypic traits of PH, RL, RDW, and SDW in response to drought stress (Supplementary Tables S1 and S2). The descriptive statistics and frequency distribution of the traits measured from the population under WW and DS conditions are presented in Table 1 and Fig. 1, respectively. Large variation was observed in all phenotypic traits in both WW and DS treatments that make the measurements suitable for GWAS analysis (Table 1).

**Table 1:** Descriptive statistics of seedling traits in 125 wheat genotypes in the well-watered and drought stressed treatments

Trait	Unit	Treatment	Min	Max	Mean	SD	CV (%)
PH	cm	WW	24.2	39.30	31.27	3.17	10.14
		DS	7.50	29.40	20.61	3.68	17.87
RL	cm	WW	60.91	146.21	102.91	18.30	17.78
		DS	23.01	108.79	58.33	16.49	28.27
RDW	g	WW	0.029	0.056	0.041	0.006	14.63
		DS	0.007	0.041	0.020	0.006	30.00
SDW	g	WW	0.043	0.101	0.067	0.011	16.42
		DS	0.007	0.069	0.034	0.010	29.41

“PH” is plant height; “RL” root length; “RDW” root dry weight; “SDW” shoot dry weight; “WW” well-watered; “DS” drought stress; “Min.” minimum; “Max.” maximum; “SD” standard deviation; “CV” coefficient of variation

The mean PH was 31.3 cm (ranging from 24.2 to 39.3 cm) in the WW treatment, whereas it was 20.6 cm (ranging from 7.5 to 29.4 cm) in the DS treatment (Fig. 1A and Table 1). For RL, mean values were 102.9 and 58.3 cm, ranging from 60.9 to 146.2 cm and from 23.0 to 108.8 cm, in the WW and DS treatments, respectively (Fig. 1B and Table 1). The mean RDW was 0.041 g (ranging from 0.029 to 0.056 g), whereas it was 0.02 g (ranging from 0.007 to 0.041 g) in the WW and DS treatments, respectively (Fig. 1C and Table 1). The mean SDW in the WW treatment was 0.067 g with a range from 0.043 to 0.101 g, while under DS the mean SDW was 0.034 g with a range from 0.007 to 0.069 g (Fig. 1D and Table 1). Overall, DS reduced PH, RL, RDW, and SDW by 34, 43, 51, and 49%, respectively. The coefficient of variation (CV) was calculated to compare the extent of variability between traits. For all traits, the CV in the DS treatment was higher than in the WW treatment. In WW, the CV ranged from 10.1% (PH) to 17.8% (RL), whereas in DS, the CV ranged from 17.9% (PH) to 30.0% (RDW) (Table 1).

The Pearson’s correlation analysis showed significant positive correlation between all traits under stress and non-stress conditions (Fig. 1E and F). The correlation coefficients between the four traits ranged from 0.43 to 0.76 in the DS treatment and from 0.23 to 0.66 in the WW treatment. In DS, the highest correlation (0.76) was between RDW and SDW following PH and SDW (0.74), while the lowest correlations were between PH and RDW (0.43) and between PH and RL (0.5) (Fig. 1E). In WW, the highest correlations were 0.66 (RDW and SDW) and 0.63 (RL and RDW), while the lowest were 0.23 (PH and RDW) and 0.38 (PH and RL) (Fig. 1F).

### Marker distribution, genetic diversity, and principal components analysis

**Fig. 2:**
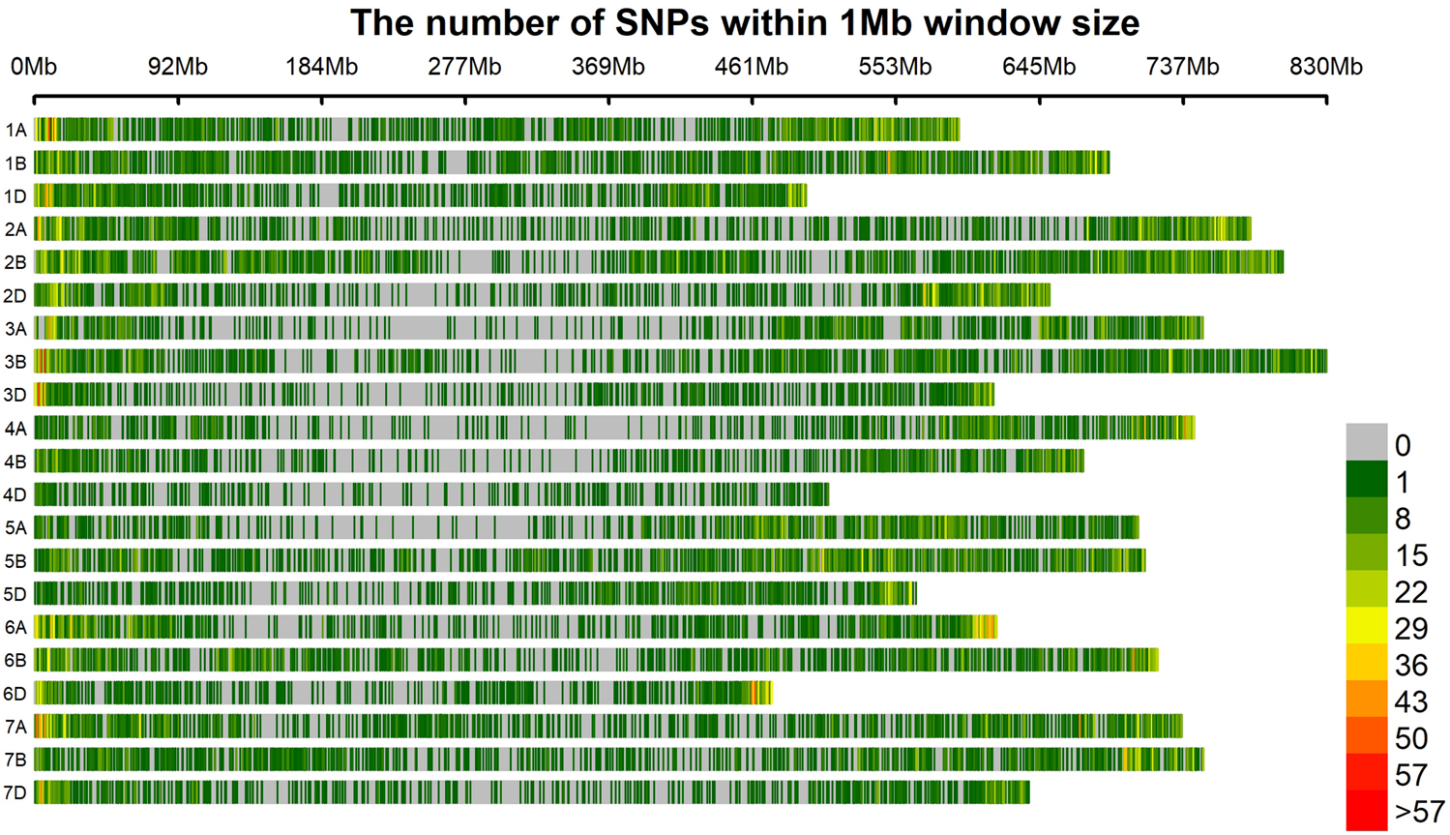
Distribution of filtered SNPs on the 21 chromosomes of wheat. The colours indicate the number of SNPs within a 1 Mb interval

After filtering, a total number of 36,586 SNP markers remained that were used for GWAS analysis. The distribution of these markers on the 21 chromosomes of a wheat is presented in Fig. 2 and Supplementary Table S3. Chromosome 2B and 1A with 3055 and 2311 SNPs and a density of 3.81 and 3.89 markers per mega-base pair had the highest number and density of markers, respectively. Chromosome 4D with 422 SNPs and a density of 0.83 markers per Mbp had the least number and density of markers. The sub-genome B had the highest number of markers (15,273 SNPs) and density (2.95 marker per Mbp), followed by sub-genome A with 13,156 SNPs and density of 2.67 markers per Mbp. The sub-genome D with 7,897 SNPs had the lowest number of markers with the least density of 2 markers per Mbp (Supplementary Table S3).

**Fig. 3:**
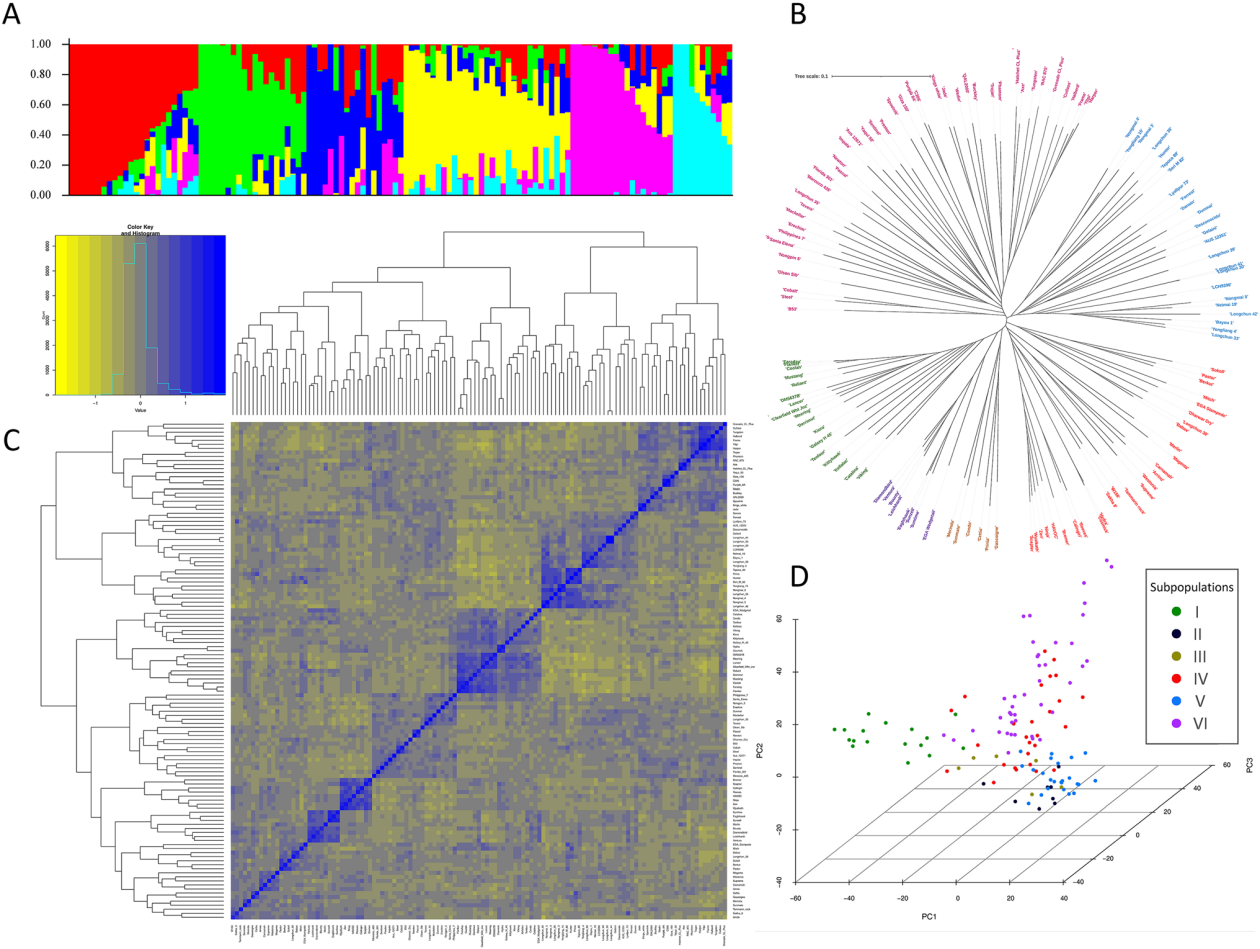
Genetic diversity of the 125 wheat accessions. (**A**) Population structure estimated by STRUCTURE with optimum sub-population (K = 6), each colour represents one subpopulation. (**B**) Phylogenetic tree, each branch indicates an accession, and the length of the branches represent the genetic distance. (**C**) Heat map of relatedness (kinship), blue colour at the middle represents the degree of relatedness. (**D**) Three-dimensional principal component analysis (PCA) plot illustrating the distribution of accessions based on the first three principal components (PC)

The Bayesian clustering analysis of population structure showed that the ΔK value reached its peak at K = 6 (Supplementary Figure S1), suggesting the entire population could be grouped into six sub-populations (Fig. 3A). The phylogenetic tree also divided the accessions into six main clusters highlighted in different colours in Fig. 3B. The kinship matrix accounts for relationships among individuals based on the degree of allele sharing. The pattern of blue shaded colour at the middle of the kinship matrix that corresponded to the degree of relatedness, indicated a stratified population structure as represented by the structure and phylogenetic analyses (Fig. 3C).

PCA was carried out to get more information about the principal structure and the first five PC scores were included as covariates for the GWAS analysis. PCA revealed that the first five principal components can explain 22.9% of the total variance among the accessions (Supplementary Table S4 and Supplementary Figure S2). The PCA chart in Fig. 3D demonstrated the first three principal components of PC1, PC2 and PC3 explained 6.8%, 5.4%, and 4.3% of the variance, respectively.

### Linkage disequilibrium (LD) and LD decay

**Fig. 4:**
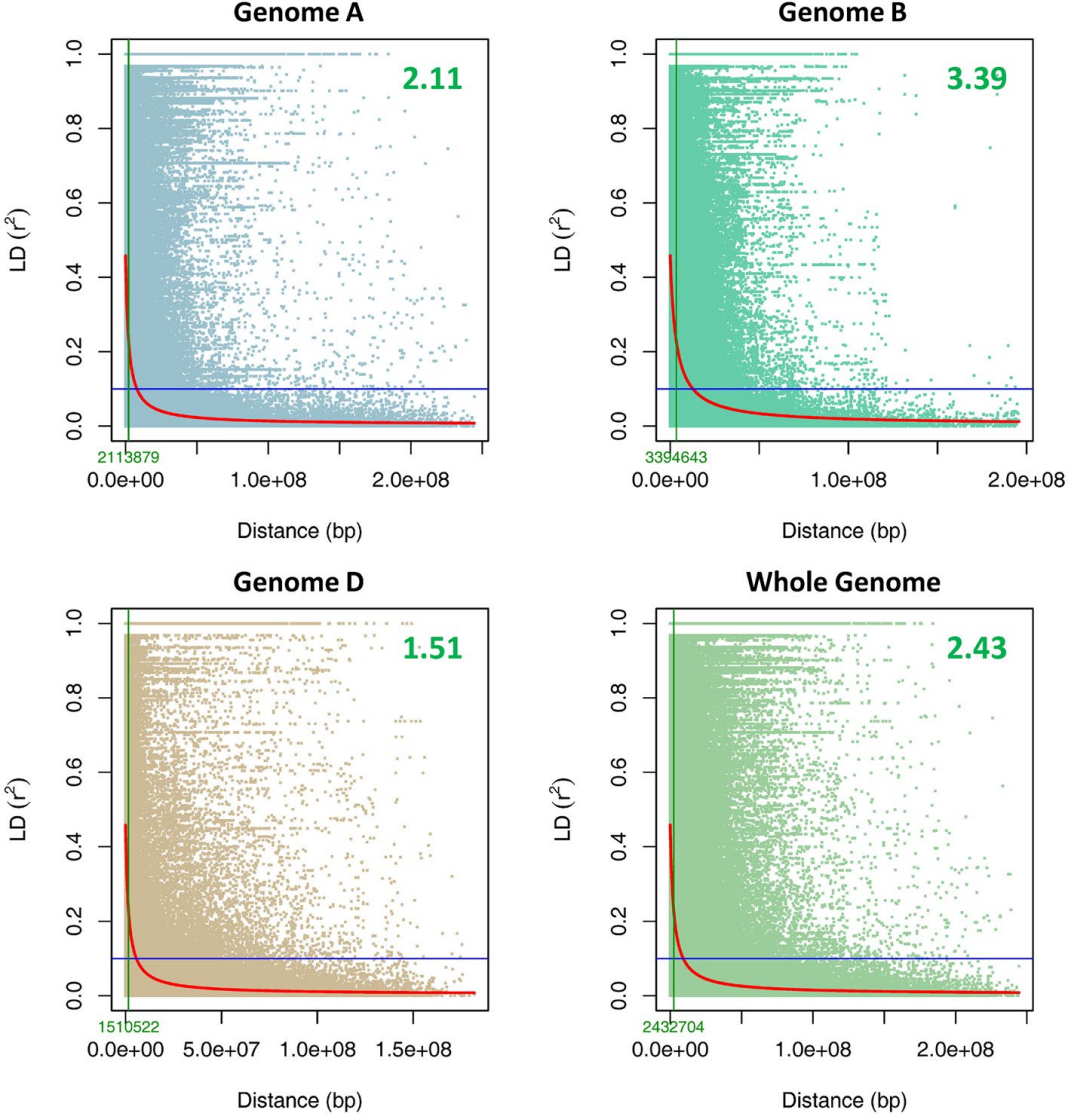
Scatter plot showing linkage disequilibrium (LD) decay in the three sub-genomes and the whole genome by plotting (*r*^2^) against genetic distance (bp) in 125 wheat accessions. The point at which LD is reduced to 50% of its maximum value is indicated by the green vertical line. LD decay at cut off point is shown by green font on the *X*-axis and with bigger font in the plots

Pair-wise LD analysis using the correlation coefficient (*r*^2^) for SNP markers showed that the rate of LD decay with genetic distance varied across different chromosomes as well as sub-genomes (Fig. 4 and Supplementary Figure S3). The LD decayed to its half at 2.43 Mb for whole genome, and 2.11 Mb for A, 3.39 Mb for B and 1.51 Mb for the D sub-genomes (Fig. 4). For individual chromosomes, chromosome 5A has the highest (4.73 Mb) and chromosome 3A had the lowest (1.07 Mb) LD decay in sub-genome A. In sub-genome B, chromosome 1B and 7B with LD decay of 6.35 and 1.64 Mb had the highest and lowest LD decay, respectively. In sub-genome D, chromosome 1D with 8.44 Mb had the highest LD decay, while chromosome 7D with 0.31 Mb had the lowest LD decay (Supplementary Figure S3).

### GWAS-revealed marker-trait associations

**Fig. 5:**
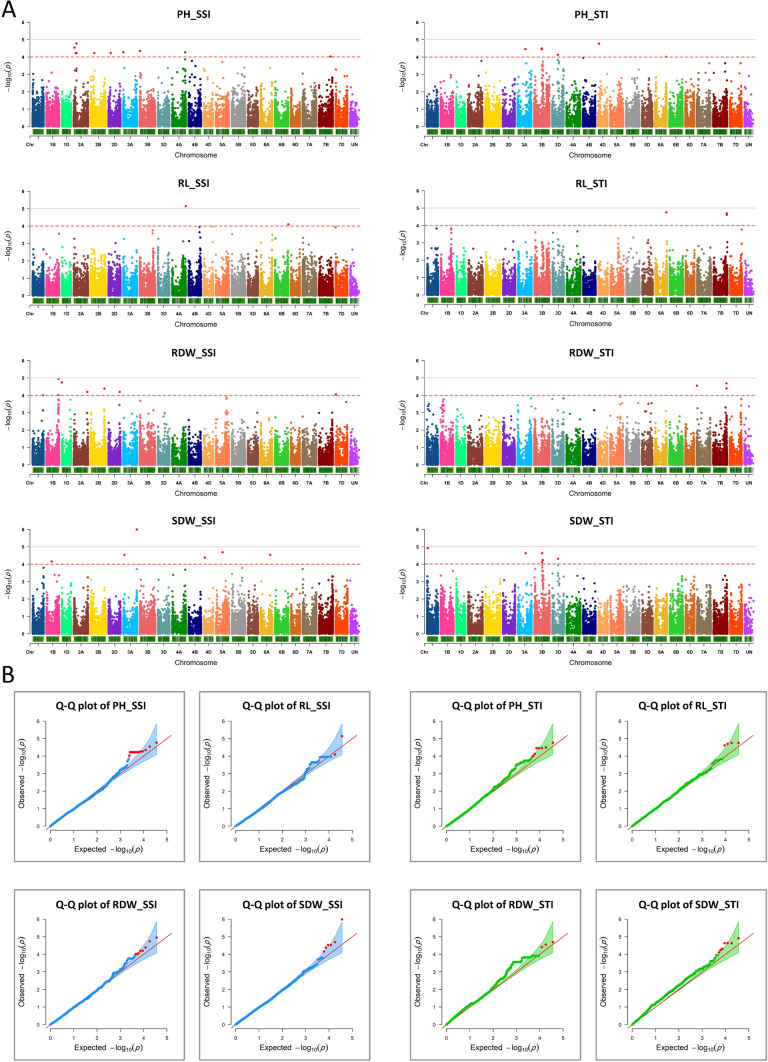
(**A**) Manhattan and (**B**) Q-Q plots of the genome-wide association (GWAS) results for stress susceptibility index (SSI, charts on left) and stress tolerance index (STI, charts on right) of seedling traits. The markers above the significant threshold value of − log10 (*p*) > 4 (red dotted line) are shown in red colour. PH, plant height; RL, root length; RDW, root dry weight; SDW, shoot dry weight

To find genomic regions significantly associated with drought tolerance, the two indices of SSI and STI were calculated for seedling traits and GWAS was performed separately for each of these indices. The Manhattan plots showing the distribution of significantly associated SNPs across the wheat genome and Q-Q plots are presented in Fig. 5A and B, respectively. Most *p*-values in the Q-Q plots were comparable to the anticipated diagonal line, suggesting that the employed GWAS model was suitable (Fig. 5B). A total of 53 SNP-trait associations were detected on 17 chromosomes using the significance threshold value of − log10 (*p*) > 4 (Supplementary Table S5). Of this number, 31 SNPs were identified for SSI and 22 were found for STI with no common SNP between the two indices. Chromosome 2A had the highest number of SNPs with 10, followed by 3B with nine SNPs. Chromosomes 1D, 5A, 6B, 7A, and 7D each had one SNP, representing the lowest number of associated markers. The R^2^ values for the 53 significant SNPs explaining phenotypic variations range from 10.8 to 25.4% (Tables 2 and 3). Of all identified SNPs, BS00086777_51 associated with RL_SSI and wsnp_BE426418A_Ta_2_1 associated with SDW_SSI had − log10 (*p*) > 5.

**Table 2:** Significant SNPs and candidate genes identified as associated with stress susceptibility indices of four drought tolerance-related traits based on GWAS of 125 wheat genotypes at the seedling stage

Trait	Marker	Chr	Allele	Marker position	log(*p*)	Gene ID	SNP-Gene distance (bp)	R^2^%	Gene function	Gene length & or. (bp)
PH_SSI	Ku_c972_560	2A	A/G	42392191	4.54	*TraesCS2A02G089200*	74	19.35	Histone-lysine N-methyltransferase ASHH2	2001−
	TA003045-1227	2A	A/G	115120841	4.22	*TraesCS2A02G162700*	Within gene	17.88	Serine/arginine rich splicing factor, putative	2310−
	Kukri_c44997_377	2A	T/C	115140823	4.22	*TraesCS2A02G162900*	Within gene	17.88	Copper-translocating P-type ATPase family protein, expressed	2461−
	wsnp_Ex_rep_c69124_68035904	2A	A/G	115421998	4.22	*TraesCS2A02G163000*	Within gene	17.88	Calmodulin-binding transcription activator	8584+
	Kukri_c7169_105	2A	A/G	138183614	4.22	*TraesCS2A02G179783*	Within gene	17.87	NA	3699−
	TA001792-1026	2A	A/G	141166706	4.78	*TraesCS2A02G181800*	Within gene	20.5	S phase cyclin A-associated protein in the endoplasmic reticulum	13583+
	TA001351-1193	2A	T/C	151258885	4.22	*TraesCS2A02G188200*	Within gene	17.88	α-1,6-mannosyl-glycoprotein 2-β-N-acetylglucosaminyltransferase	11695+
	GENE-1181_262	2A	A/G	151263814	4.22	*TraesCS2A02G188200*	Within gene	17.88	α-1,6-mannosyl-glycoprotein 2-β-N-acetylglucosaminyltransferase	11695+
	Excalibur_c27210_183	2A	T/G	152010707	4.22	*TraesCS2A02G188400*	Within gene	17.88	Zinc finger protein-like protein	3815+
	BobWhite_c32226_104	2B	T/C	180822839	4.22	*TraesCS2B02G201800*	Within gene	17.89	Pentatricopeptide repeat-containing protein, putative	3426−
	GENE-2601_393	2D	T/C	122703750	4.22	*TraesCS2D02G179100*	Within gene	17.88	Methyltransferase-like protein	3311−
	Kukri_rep_c89183_282	3A	T/C	8686047	4.27	*TraesCS3A02G009600*	Within gene	18.11	U-box domain-containing protein	4780+
	Excalibur_rep_c104498_168	3B	A/G	16444577	4.35	*TraesCS3B02G034400*	Within gene	18.45	WD repeat-containing protein 1	5163+
	Excalibur_c7753_51	4A	A/G	707041300	4.27	*TraesCS4A02G437200*	Within gene	18.08	Protein ENHANCED DISEASE RESISTANCE 2-like	7934−
	GENE-4701_272	7B	A/G	626112422	4.03	*TraesCS7B02G363700*	Within gene	13.82	3-hydroxyisobutyrate dehydrogenase	3244−
RL_SSI	BS00086777_51	4A	A/G	739067906	5.14	*TraesCS4A02G485400*	Within gene	15.67	Acid invertase 1	3823+
	Tdurum_contig65998_182	6B	A/G	717967277	4.10	*TraesCS6B02G469000*	Within gene	12.36	PRLI-interacting factor A	2255−
RDW_SSI	IACX5803	1A	A/C	563180048	4.01	*TraesCS1A02G397500*	Within gene	12.94	Ubiquitin	730−
	Excalibur_c49496_705	1B	T/C	652453983	4.01	*TraesCS1B02G426200*	Within gene	12.94	RING/U-box superfamily protein	4769+
	IAAV565	1B	T/C	652457867	4.95	*TraesCS1B02G426300*	Within gene	16.13	Ras-related protein	1690−
	wsnp_JD_c5316_6447231	1D	A/G	2077073	4.74	*TraesCS1D02G003900*	Within gene	18.12	Peroxisomal membrane protein PEX14	3921−
	tplb0046b02_1751	2A	T/C	734887845	4.2	*TraesCS2A02G508200*	Within gene	13.6	Sulfate transporter	6002+
	BS00065327_51	2B	T/G	731481895	4.39	*TraesCS2B02G535600*	Within gene	14.24	3-ketoacyl-CoA synthase	1690−
	wsnp_RFL_Contig1892_1042675	2D	T/C	601600198	4.2	*TraesCS2D02G508800*	Within gene	13.6	Sulfate transporter	5947+
	Kukri_c22953_855	7D	A/G	18000929	4.05	*TraesCS7D02G035000*	496	13.1	NBS-LRR disease resistance protein	2647−
SDW_SSI	BobWhite_c35311_122	1B	T/C	286044097	4.16	*TraesCS1B02G163800*	7845	13.89	Photosystem I P700 chlorophyll a apoprotein A1	1652+
	Excalibur_c10198_691	3A	A/C	64324895	4.54	*TraesCS3A02G099900*	9136	15.44	Cytochrome P450, family 88, subfamily A, polypeptide 3	465−
	wsnp_BE426418A_Ta_2_1	3A	T/C	725738598	5.99	*TraesCS3A02G502300*	Within gene	25.42	NAD(P)H:plastoquinone dehydrogenase complex subunit O	1626+
	tplb0037i09_749	4D	A/G	51309211	4.38	*TraesCS4D02G077100*	5424	14.81	OBF binding protein 1	661−
	CAP7_c6648_230	5A	A/G	353989255	4.69	*TraesCS5A02G165400*	Within gene	16.08	Ribulose bisphosphate carboxylase small chain	1371+
	BobWhite_c9670_25	6A	T/C	492805334	4.54	*TraesCS6A02G267300*	Within gene	15.44	Phosphoribulokinase	2360+

“PH” is plant height; “RL” root length; “RDW” root dry weight; “SDW” shoot dry weight; “SSI” stress susceptibility index; “Chr.” chromosome; “bp” base pair. “R^2^” is the phenotypic variation explained by each marker. “Or.” is the gene’s orientation that represents on which strand the genes are located. The (+) represents the forward strand, whereas the (−) represents the complementary strand

**Table 3:** Significant SNPs and candidate genes identified as associated with stress tolerance indices of four drought tolerance-related traits based on GWAS of 125 wheat genotypes at the seedling stage

Trait	Marker	Chr	Allele	Marker position	log(*p*)	Gene ID	SNP-Gene distance (bp)	R^2^%	Gene function	Gene length & or. (bp)
PH_STI	Kukri_c40909_784	3A	T/C	423958992	4.45	*TraesCS3A02G226500*	Within gene	12.66	Auxilin-related protein 1	15337+
	Excalibur_c63730_660	3B	T/C	416279752	4.49	*TraesCS3B02G258600*	Within gene	12.79	Polygalacturonase	4667+
	Tdurum_contig28384_102	3B	A/G	416283216	4.45	*TraesCS3B02G258700*	Within gene	12.66	Peptide deformylase	4797−
	Kukri_rep_c75974_432	3B	A/C	416799894	4.45	*TraesCS3B02G259100*	5942	12.66	Receptor-like kinase	5116−
	RAC875_c17404_1160	3D	A/G	305863726	4.13	*TraesCS3D02G224500*	Within gene	11.69	Auxilin-related protein 1	16040+
	wsnp_Ex_c683_1341113	4D	A/G	54447007	4.77	*TraesCS4D02G080400*	27391	13.61	B3 domain-containing protein	2171+
	BS00003185_51	6A	A/G	596165675	4.02	*TraesCS6A02G372300*	Within gene	11.35	Methyltransferase-like protein	3186+
RL_STI	GENE-4268_101	6A	A/C	585192104	4.75	*TraesCS6A02G353500*	Within gene	13.55	Kinase	7573+
	IACX473	6A	A/C	585192973	4.75	*TraesCS6A02G353500*	Within gene	13.55	Kinase	7573+
	BS00075300_51	7B	T/C	713379927	4.61	*TraesCS7B02G452000*	74089	13.13	Pm3-like disease resistance protein	5229−
	BS00075299_51	7B	T/C	713379957	4.7	*TraesCS7B02G452000*	74059	13.37	Pm3-like disease resistance protein	5229−
RDW_STI	Excalibur_c35183_406	7A	T/C	8694709	4.56	*TraesCS7A02G021400*	Within gene	14.76	NBS-LRR-like resistance protein	4838−
	BS00075300_51	7B	T/C	713379927	4.69	*TraesCS7B02G452000*	74089	15.21	Pm3-like disease resistance protein	5229−
	BS00075299_51	7B	T/C	713379957	4.4	*TraesCS7B02G452000*	74059	14.23	Pm3-like disease resistance protein	5229−
SDW_STI	RAC875_c24163_501	1A	T/C	32546271	4.92	*TraesCS1A02G051100*	Within gene	13.6	F-box/LRR-repeat protein	2641+
	Kukri_c40909_784	3A	T/C	423958992	4.63	*TraesCS3A02G226500*	Within gene	12.77	Auxilin-related protein 1	15337+
	Kukri_rep_c70097_286	3B	T/G	415930394	4.11	*TraesCS3B02G258000*	Within gene	11.27	Auxilin-related protein 1	12142−
	Excalibur_c63730_660	3B	T/C	416279752	3.95	*TraesCS3B02G258600*	Within gene	10.79	Polygalacturonase	4667+
	Tdurum_contig28384_102	3B	A/G	416283216	4.63	*TraesCS3B02G258700*	Within gene	12.77	Peptide deformylase	4797−
	Kukri_rep_c75974_432	3B	A/C	416799894	4.63	*TraesCS3B02G259100*	5942	12.77	Receptor-like kinase	5116−
	RFL_Contig2538_204	3B	T/C	455681347	4.24	*TraesCS3B02G284600*	Within gene	11.63	Regulator of Vps4 activity in the MVB pathway protein	5372−
	Excalibur_c8871_1890	3D	T/C	301442744	4.31	*TraesCS3D02G220900*	Within gene	11.85	Elongation factor	5712−

“PH” is plant height; “RL” root length; “RDW” root dry weight; “SDW” shoot dry weight; “STI” stress tolerance index; “Chr.” chromosome; “bp” base pair. “R^2^” is the phenotypic variation explained by each marker. “Or.” is the gene’s orientation that represents on which strand the genes are located. The (+) represents the forward strand, whereas the (−) represents the complementary strand

Regarding individual traits, 22 SNP markers on 10 chromosomes were associated with PH; 15 of these were found for PH_SSI and 7 for PH_STI. Chromosome 2A, 3B, and 3A had nine, four, and two SNPs respectively and chromosome 2B, 2D, 3D, 4A, 4D, 6A, and 7B had one SNP each (Fig. 5A and Tables 2 and 3). Six markers were found for RL, including two SNPs located on chromosome 4A and 6B for RL_SSI and four markers on 6A and 7B for RL_STI. Of the 11 SNPs associated with RDW, eight were identified for RDW_SSI and three were identified for RDW_STI by the GWAS analysis. Two of these SNPs were located on 1B, two on 7B, and one on each of 1A, 1D, 2A, 2B, 2D, 7A, and 7D chromosomes. For SDW, 14 markers (six markers for SDW_SSI and eight for SDW_STI) were associated, including five SNPs located on 3B, three on 3A, and one on each 1A, 1B, 3D, 4D, 5A, and 6A (Fig. 5A and Tables 2 and 3).

### Identified candidate genes

Forty-four unique candidate genes were identified for the four drought-tolerance related traits at the seeding stage based on the position of the associated SNP markers (Tables 2 and 3). Of this number, 30 unique genes were found for SSI and 14 for STI. For 36 of the identified genes, the SNP markers were located within the genes, while for eight of the genes, markers were 74 to 74,059 base pairs away from the gene. The length of the genes was different and ranged from 465 to 16,040 base pairs. Regarding the gene orientation, 21 of the genes were located on the forward strand (+) whereas the rest of the 23 genes were on the complementary strand (−) (Tables 2 and 3).

**Fig. 6:**
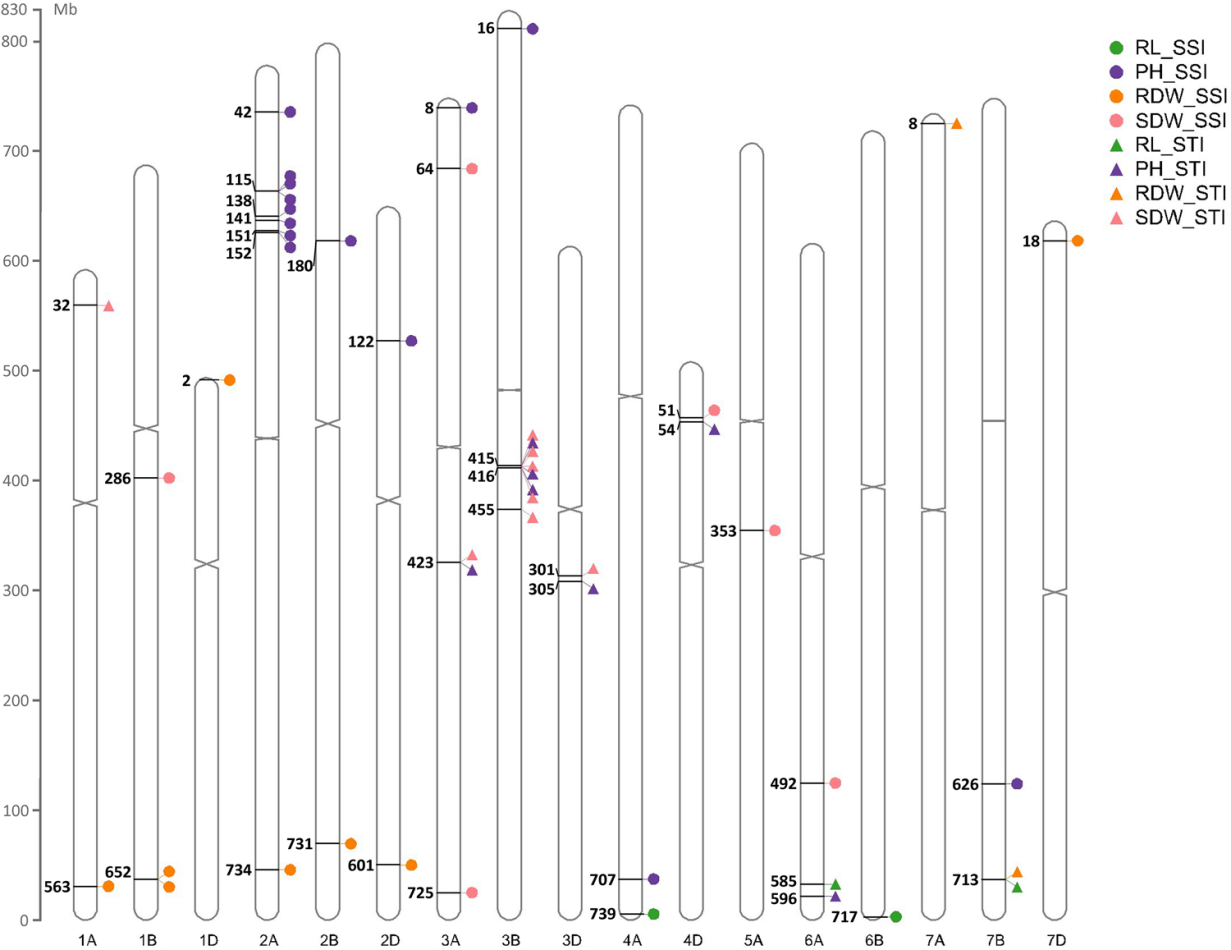
The distribution of the identified candidate genes of stress susceptibility index (SSI) and stress tolerance index (STI) for plant height (PH), root length (RL), root dry weight (RDW), and shoot dry weight (SDW) on 17 wheat chromosomes. Numbers indicate the physical positions (Mb) of the genes on the chromosomes

Some of the genes were common between different traits. *TraesCS7B02G452000* that encodes the Pm3-like disease-resistance protein was found for both RL_STI and RDW_STI. The highest number of common genes were shared between PH_STI and SDW_STI including *TraesCS3B02G258600*, *TraesCS3A02G226500*, *TraesCS3B02G258700*, and *TraesCS3B02G259100* that encoded polygalacturonase, auxilin-related protein 1, peptide deformylase, and receptor-like kinase, respectively (Tables 2 and 3). The distribution of the 44 identified genes on the 17 chromosomes of wheat is illustrated in Fig. 6. All genes associated with RDW_SSI and RDW_STI were located at the very beginning of the short arms of 1D, 7A, and 7D or within 70 Mbp of the end of chromosomes 1A, 1B, 2A, 2B, 2D, and 7B. Similarly, the genes for RL_SSI and RL_STI were located at very end of the chromosomes 4A, 6A, 6B, and 7B. Most of the genes associated with PH_SSI were on the short arms of 2A, 2B, 2D, 3A, and 3B chromosomes with a cluster of seven genes at 115–152 Mbp of the 2AS. Four of the genes related to SDW_STI (three of them were common between PH_STI and SDW_STI) tended to cluster together at 415–455 Mbp of the 3BL (Fig. 6).

### Expression of the candidate genes in tolerant and susceptible genotypes

The expression of the 44 identified candidate genes in the leaves and roots of drought tolerant (Zubkov) and susceptible (Atay85) wheat cultivars are presented in Supplementary Figure S4 and S5. In the leaves in the WW treatment, there were minimal differences in the expression of nearly all candidate genes between tolerant and susceptible genotypes (average relative expression difference of 0.32 for all genes) (Supplementary Figure S4A). However, in the DS treatment, the majority of genes exhibited increased expression in the leaves of the tolerant genotype and decreased expression in the susceptible genotype. The average relative expression difference between tolerant and susceptible genotypes increased to 0.67 in the DS treatment, with 35 genes up-regulated and 3 down-regulated in the tolerant genotype relative to the susceptible genotype (Supplementary Figure S4B). Among the genes, *TraesCS4A02G485400*, encoding acid invertase 1, showed the highest relative increase in expression in the tolerant genotype compared to the susceptible genotype in the DS treatment. This was followed by *TraesCS2A02G188200* and *TraesCS2A02G181800*, which encode α-1,6-mannosyl-glycoprotein 2-β-N-acetylglucosaminyltransferase and S phase cyclin A-associated protein, respectively.

In roots in the WW treatment, the candidate genes, on average, exhibited higher expression levels in the susceptible genotype compared to the tolerant genotype (33 genes down-regulated and 6 up-regulated in the tolerant genotype relative to the susceptible genotype in the WW treatment) (Supplementary Figure S5A). Conversely in the DS treatment, the greater increase in gene expression in the tolerant genotype led to more comparable expression levels between the tolerant and susceptible genotypes during stress. The average relative expression difference between the tolerant and susceptible genotypes decreased from 0.43 in the WW to 0.19 in the DS treatment (Supplementary Figure S5B).

## Discussion

In this study we evaluated four phenotypic traits among 125 accessions under WW and DS conditions. In the accession panel used in this study, significant phenotypic variation was observed among lines for all seedling drought tolerance-related traits in both the WW and DS treatments (Table 1 and Supplementary Table S1). This suggests that the genotypes used in this study were a good genetic source for drought tolerance research. This result agreed with a previous study by Liu et al. (2022) who found abundant phenotypic variation for wheat yield traits through evaluation of the same panel in six rainfed environments. In this study, the CV for all four traits was higher in the DS compared to the WW treatment (Table 1). Similarly, the higher variation for phenotypic traits has previously been reported under drought stress conditions in wheat and cotton (Hou et al. 2018; Grzesiak et al. 2019). In comparison with WW plants, drought stress significantly reduced the average of all four PH, RL, RDW, and SDW traits. It is well known that drought stress at the vegetative stage can negatively impact morphological, physiological, and biochemical dynamics in plants and reduce phenotypic traits such as shoot length, shoot dry weight, root volume, root length, and root dry weight (Seleiman et al. 2021).

Correlation analysis showed that all the traits were positively co-related with each other (Fig. 1E and F). The significant highly positive correlations between RDW_SDW, PH_SDW, and RL_RDW in both the WW and DS treatments indicated the possibility of simultaneous improvement of these traits. This finding was in line with a previous report on a diverse panel of wheat genotypes that was studied in order to improve drought tolerance (Danakumara et al. 2021). The SDW is an easily measurable trait and its high correlation with RDW suggests that it should be valuable in providing a general idea about the development of the root system at the seedling stage. The strong correlation between RDW_SDW might be due to the importance of roots in supplying water and nutrients to the shoots (Zhao et al. 2019).

The LD decay was used to determine the density of marker coverage required for GWAS analysis. For faster LD decay, a higher density of markers is needed to capture the markers close enough to the causal loci (Flint-Garcia et al. 2003). In this study the LD decay was 2.43 for the whole genome, 2.11 for A, 3.39 for B, and 1.51 for D sub-genomes (Fig. 4). A similar LD pattern for the whole genome, sub-genomes A, B, and D has been reported for the same population in a previous study (Kurya et al. 2022). A faster LD decay in the D genome, comparable to A and B genomes was also observed in wheat pre-breeding lines (Ledesma-Ramírez et al. 2019). In agreement with our result, an average of ~ 2 Mb was observed for whole genome LD decay in a set of CIMMYT spring bread wheat lines (Sehgal et al. 2020). However, in contrast, the slowest LD decay in the D genome, compared to A and B genomes, has been reported in previous studies (Chao et al. 2007; Liu et al. 2017). Contrary to the fast LD decay that was witnessed in this study for the whole genome, the slower LD decay distances have been observed in a set of hexaploid wheat collections from Kazakhstan (22 Mb) and Mexican bread wheat landraces (23 Mb) (Kokhmetova et al. 2021; Vikram et al. 2021). The variation in the LD decay among various GWAS populations may be due to different factors including mutation, selection, size of the population, recombination frequency, genetic drift, admixtures, pollination behaviour and non-random mating (Vos et al. 2017). The faster LD decay suggests high levels of genetic diversity in the mapping population used in this study, which consists of lines selected from a wide range of genetic backgrounds. The high diversity of this panel has also been observed in previous studies (Kurya et al. 2022; Liu et al. 2022).

STI and SSI are two widely employed criteria for evaluation of accessions in plant abiotic stress studies. It has been reported that STI has greater efficiency in identifying tolerant genotypes, while SSI is more effective for selection of sensitive genotypes under drought stress conditions (Ghaffari et al. 2012). Using both indices at the same time for a panel can deliver a more accurate assessment of drought tolerance (Wu et al. 2022). The genotypes with high STI and low SSI values are considered drought tolerant in wheat (Ayed et al. 2021). A total of 53 SNP markers were identified associated with the four phenotypic traits, 31 of them were found using SSI, and 22 were detected using STI, with no common SNP between the two indices.

In this study, the − log10 (*p*) > 4 was used to indicate significant marker-trait association because the Bonferroni threshold (− log10 (*p*) > 5.86) was too strict. The complex and polygenic nature of the traits, where weaker genetic signals may collectively contribute to variation, provides a potential explanation (Liu et al. 2022). Additionally, limitations stemming from the size and highly structured nature of our population may contribute to detecting weaker signals (Wang and Xu 2019). Navigating the trade-off between sensitivity and specificity, we selected a less strict threshold to enhance sensitivity while mitigating the risk of false negatives. This choice aligns with practices in comparable studies and is further supported by the consideration of biological plausibility (Kurya et al. 2022; Liu et al. 2022). Associations identified using less stringent thresholds are considered preliminary, so we recommend rigorous follow-up analyses and validation steps, including assessments in independent datasets and functional studies, to ensure a comprehensive validation process for the identified candidate markers.

The significant SNPs identified in this investigation explained between 10.8% and 25.4% of the phenotypic variation and are considered major QTLs (Elattar et al. 2021). Many of these loci are located towards the telomere ends of chromosomes (Fig. 6) that are known to be gene-rich regions (See et al. 2006). In support of our findings, the genetic dissection of the seedling root system of wheat for improved drought tolerance has revealed associated markers on 558 Mb of chromosome 1A for average root diameter; 10 Mb of 1D for root number and root length; 725 Mb of 4A for root length, root volume, average root diameter, and lateral root density; 599, 600, and 611 Mb of 6A for lateral root number, lateral root size, average diameter, seminal root number, and root volume; 710 Mb of 6B for root length and root volume; 591, 674, and 675 Mb of 7B for shoot dry weight, lateral root density, and root length (Danakumara et al. 2021). A QTL meta-analysis to discover consensus genomic regions in wheat for root-related traits reported seven QTLs on approximately 583 Mb of 1A, two on ~ 661 Mb of 1B, three on ~ 19 Mb of 1D, 17 on ~ 627 Mb of 2B, seven on ~ 570 Mb of 2D, five on ~ 702 Mb of 4A, six on ~ 613 Mb of 6A, four on ~ 673 Mb of 6B, and 18 on ~ 728 Mb of 7B (Soriano and Alvaro 2019).

In this study, twenty candidate genes were found to be related with plant height, including 14 genes related to PH_SSI and 6 to PH_STI (Tables 2 and 3). For PH_SSI, S phase cyclin A-associated protein in the endoplasmic reticulum (SCAPER) explained the highest variation (R^2^ = 20.5) followed by Histone-lysine N-methyltransferase ASHH2 (R^2^ = 19.4) (Table 2). SCAPER is an ER-localised protein that is involved in the regulation of cell cycle proliferation and cell expansion (controls the S-to-M phase) by generating cyclin A2 in the cytoplasm (Tsang et al. 2007). Histone methylation, which is mediated by histone lysine methyltransferases, is a mechanism associated with gene expression regulation and has been reported to be involved in plant stress memory under drought, heat, cold, salinity, and dark (Zhou et al. 2020). WD repeat-containing protein 1 (WDR1) plays an important role in plant development by dynamic reorganization of the actin cytoskeleton (Ono 2018). In rice, overexpression or RNA interference of WDR1 reduced the size of plants (Shi et al. 2013). Plant U-box (PUB) proteins are ubiquitin ligases (E3) involved in different functions in plant development and stress responses including hormone signalling [e.g., abscisic acid (ABA)], cell death, senescence, and plant survival following stress or pathogen attack (Vogelmann et al. 2014). The pentatricopeptide repeat (PPR) proteins play roles in plant growth and development as well as various biotic and abiotic stresses (Xing et al. 2018). In *Arabidopsis*, the mutant for PPR proteins known as Slow Growth 1 (*slg1*) and *slo2* affected shoot growth, ABA signalling, and drought stress tolerance (Yuan and Liu 2012; Lv et al. 2014). The serine/arginine-rich splicing factors (SRs) that generate proteome diversity through the splicing process of the precursor RNA, provides a key mechanism in regulating gene expression during development and stress responses (Duque 2011). Calmodulin-binding transcription activator (CAMTA) and zinc finger protein-like protein (ZNF) as members of TFs are associated with plant growth, development, and response to stresses (Han et al. 2020; Yang et al. 2022). Alpha-1,6-mannosyl-glycoprotein 2-β-N-acetylglucosaminyltransferase has a critical role in protein N-glycosylation and has been reported to be involved in plant growth and development under stress conditions (Yoo et al. 2021). Under metabolic stress conditions, amino acid catabolism which is conducted by 3-hydroxyisobutyrate dehydrogenase can be an alternative substrate for production of adenosine triphosphate (ATP) by respiration (Schertl et al. 2017).

Regarding PH_STI (Table 3), the B3 domain-containing protein is a TF responsive to ABA and auxin phytohormones that engage in developmental processes such as plant growth (Waltner et al. 2005). Polygalacturonase (PG) is a hydrolase enzyme that degrades cell wall pectin that plays a role in plant organ senescence and abiotic stress responses (Yang et al. 2018). Liu et al. (2014) have shown that the overexpression of the β subunit of PG1 in rice reduced pectin content and cell adhesion and increased abiotic stress sensitivity. Peptide deformylase is an important enzyme required for the removal of the N-formyl group from newly translated proteins and is necessary for N-terminal protein processing (Hou et al. 2007). Receptor-like kinases (RLKs) are the largest gene family in plants and play a role in plant development and abiotic stress responses to drought, cold, and salt (Ye et al. 2017). From different genes on different chromosomes, methyltransferase-like (METTL) protein was shown to be important for both PH_SSI and PH_STI. The methylation of DNA, RNA, and proteins by METTL leads to epigenetic and epitranscriptomic regulation of numerous biological processes as well as regulation of gene expression (Wong and Eirin-Lopez 2021). Functional analysis of the protein arginine methyltransferase (*ZmPRMT1*) showed it has critical roles in abiotic stress tolerance in *Arabidopsis* (Ling et al. 2022).

For root length, two candidate genes were identified for RL_SSI and two for RL_STI (Tables 2 and 3). Acid invertase 1 catalyses the hydrolysis of sucrose into fructose and glucose which can enhance plant tolerance against abiotic stresses such as drought, salt, heat and cold through osmotic regulation, regulation of stomatal conductance, and maintenance of energy homoeostasis (Ruan et al. 2010). Kim et al. (2000) showed that enhanced unloading and vascular accumulation of glucose and fructose due to a stronger induction of vacuolar acid invertase activity in primary roots compared to young leaves, can be a reason for the higher root-to-shoot dry weight ratio under water stress in maize. PKs catalyse the reversible transfer of the γ-phosphate of ATP to phosphorylate serine, threonine, or tyrosine residues in protein and modify their activity. PKs by stress sensing and signal transduction have a key role in plant responses to different stresses such as drought, salt, and cold (Chen et al. 2021b). The regulation of root growth through the interaction of ABA with various PKs such as SnRK1, SnRK2.2 and SnRK2.3 have been reported in other studies (Fujii et al. 2007; Belda-Palazón et al. 2022).

Nine different candidate genes were identified for RDW_SSI and RDW_STI (Tables 2 and 3). It has been reported that a Ras-related small GTP-binding protein (RabE1c) by mediating the interaction with ABA receptors, adjusts stomatal movements and drought tolerance (Chen et al. 2021a). Peroxins such as PEX14 play an important role in the biogenesis of peroxisomes by participating in the import machinery for peroxisomal membrane proteins (PMPs) and matrix proteins into the organelles (Wang et al. 2019a). It is evident that very long-chain fatty acids (VLCFA) that are catalysed by a 3-ketoacyl-CoA synthase 4 (KCS4) are essential for root and pollen tube growth (Kim et al. 2021). Sulfate transporters manage the transport of SO_4_^−2^ as an essential factor for drought stress responses in plants. Sulfate is required for the synthesis of cysteine, which is essential for ABA synthesis (Gallardo et al. 2014). Ubiquitin-small subunit ribosomal protein S27Ae and RING/U-box superfamily protein both belong to the ubiquitin system. The U-box proteins function as E3 ligases that engaged in various biological processes, such as plant stress response through protein degradation and post-translational modification (Wang et al. 2020b). Some disease resistance proteins such as NBS-LRR and Pm3-like disease resistance proteins have been identified for RDW_SSI and RDW_STI. The interaction between drought stress and powdery mildew infection has been reported in tomato (Sunarti et al. 2022). The improved drought tolerance by increased expression of the CC–NBS–LRR protein encoding gene (*ADR1*) in *Arabidopsis* suggested possible overlap between disease resistance and drought tolerance signalling networks (Chini et al. 2004).

For SDW, six different candidate genes were identified for SDW_SSI (Table 2) and seven for SDW_STI (Table 3). Ribulose-1,5-bisphosphate carboxylase (RuBisCO) and phosphoribulokinase (PRK), two key enzymes of the photosynthetic Calvin cycle, play essential roles in regulation of photosynthesis in plants (Kono et al. 2017). The chloroplast NAD(P)H dehydrogenase complex is essential for plant growth and development during stress periods by facilitating cyclic electron transport in the thylakoid membranes (Ma et al. 2021a). Cytochrome P450 (CYP88A) plays a key role in gibberellin synthesis that is involved in stem elongation and other plant development (Helliwell et al. 2001). OBF binding protein 1 is a plant TF that plays a role in the regulation of gene expression in response to various environmental stressors, such as light, temperature, and drought (Samtani et al. 2022). Photosystem I P700 chlorophyll a apoprotein A1 is a molecule that is bound to chlorophyll a and plays a crucial role in the stability and regulation of the photosynthetic light reactions in plants (Eichacker et al. 1996). F-box proteins helps the cell to keep its protein homeostasis under stress conditions by targeting specific proteins for degradation (Hong et al. 2021). Elongation factors are proteins involved in the process of protein synthesis in cells also known as translation (Xu et al. 2022). Vps4 is involved in the final stages of MVB formation, a type of endosome that contains intraluminal vesicles and is involved in degradation of damaged proteins and lipids, which helps to maintain cellular homeostasis and prevent cellular damage (Wang et al. 2020a). Auxilin-related protein 1, peptide deformylase, a receptor-like kinase, and polygalacturonase were shown to control SDW_STI and were also found to control PH_STI (Table 3). The role of Auxilin-related protein 1 in plant stress responses is not well understood, but likely to be involved in the regulation of clathrin-mediated endocytosis and actin dynamics, which are both important for plant stress responses (Schwihla and Korbei 2020).

## Conclusion

This study has improved our understanding of the genetic basis for drought tolerance traits in wheat seedlings, shedding light on key traits such as plant height, root length, and root and shoot dry weight. The identification of 53 SNPs associated with stress susceptibility and tolerance indices for these traits, particularly on chromosomes 2A and 3B, opens new avenues for targeted genetic improvement strategies. The 44 unique candidate genes unveiled, with their diverse roles in plant growth, development, and stress responses, present a valuable resource for future investigations and breeding programs. The clustering pattern observed, especially regarding genes associated with SSI of plant height and STI of plant height and shoot dry weight, offers specific genomic regions worthy of further exploration. Looking ahead, our focus will shift towards functional validation studies and the integration of these candidate genes into breeding programs aimed at enhancing wheat drought tolerance. This research not only contributes to our fundamental knowledge of wheat biology but also holds practical implications for crop improvement in the face of increasing climate challenges, marking a crucial step towards sustainable agriculture.

### Supplementary Information

Below is the link to the electronic supplementary material.

Supplementary file1 (XLSX 4194 KB)

## Data Availability

The phenotypic data supporting the findings of this study are available in Supplementary Table S1. The 90K SNP array genotypic data can be obtained upon request from the corresponding authors.

## Abbreviations

ABA: Abscisic acid
ANOVA: Analysis of variance
ATP: Adenosine triphosphate
CAMTA: Calmodulin-binding transcription activator
CTAB: Cetyl trimethyl ammonium bromide
CV: Coefficient of variation
DS: Drought stress
ER: Endoplasmic reticulum
GWAS: Genome-wide association study
IWGSC: The International Wheat Genome Sequencing Consortium
KCS4: 3-Ketoacyl-CoA synthase 4
LD: Linkage disequilibrium
LEA: Late embryogenesis abundant proteins
MAF: Minor allele frequency
MCMC: Markov-Chain Monte Carlo
METTL: Methyltransferase-like
MLM: Mixed linear model
NADP +: Nicotinamide adenine dinucleotide phosphate
PC: Pot capacity
PCA: Principle components analysis
PG: Polygalacturonase
PH: Plant height
PKs: Protein kinases
PMPs: Peroxisomal membrane proteins
PPR: Pentatricopeptide repeat
PRK: Phosphoribulokinase
PUB: Plant U-box
QTL: Quantitative trait locus
RCBD: Randomized complete block design
RDW: Root dry weight
RILs: Recombinant inbred line populations
RL: Root length
RLKs: Receptor-like kinase
RuBisCO: Ribulose-1,5-bisphosphate carboxylase
SCAPER: S phase cyclin A-associated protein in the endoplasmic reticulum
SDW: Shoot dry weight
SNP: Single nucleotide polymorphism
SRs: Serine/arginine-rich splicing factors
SSI: Stress susceptibility index
STI: Stress tolerance index
TFs: Transcription factors
TRs: Transcriptional regulators
VLCFA: Very long-chain fatty acids
WDR1: WD repeat-containing protein 1
WW: Well-watered
ZNF: Zinc finger protein-like protein
